# Effect of Gamma Irradiation on the Antibody Response Measured in Human Serum from Subjects Vaccinated with Recombinant Vesicular Stomatitis Virus–Zaire Ebola Virus Envelope Glycoprotein Vaccine

**DOI:** 10.4269/ajtmh.19-0076

**Published:** 2019-06-03

**Authors:** Rebecca J. Grant-Klein, Joseph Antonello, Rick Nichols, Sheri Dubey, Jakub Simon

**Affiliations:** 1 Merck & Co., Inc., Kenilworth, New Jersey;; 2 Crozet BioPharma, Devens, Massachusetts

## Abstract

rVSVΔG-ZEBOV-GP vaccine is a live recombinant (r) vesicular stomatitis virus (VSV), where the VSV G protein is replaced with the Zaire Ebola virus (ZEBOV) glycoprotein (GP). For vaccine immunogenicity testing, clinical trial sera collected during an active ZEBOV outbreak underwent gamma irradiation (GI) before testing in biosafety level 2 laboratories to inactivate possible wild-type ZEBOV. Before irradiating pivotal trial samples, two independent studies evaluated the impact of GI (50 kGy) on binding ZEBOV-GP (ELISA) antibodies against rVSVΔG-ZEBOV-GP, using sera from a North American phase 1 study. Gamma irradiation was associated with slightly higher antibody concentrations in pre-vaccination samples and slightly lower concentrations postvaccination. Results indicate that GI is a viable method for treating samples from regions where filoviruses are endemic, with minor effects on antibody titers. The impact of GI on immunogenicity analyses should be considered when interpreting data from irradiated specimens.

## INTRODUCTION

With the encroachment of humans into tropical rain forests and international transport becoming more widely available, there has been an increase in emerging infectious diseases.^1^ This trend is likely to increase over the coming decades. Vaccines aimed at preventing highly lethal diseases are challenging to evaluate because the biological specimens may contain contagious and lethal pathogens that are not allowed in standard regulated clinical laboratories, which are typically only equipped to handle biosafety level (BSL)-2 agents. Safety concerns require that additional precautions are implemented in the laboratories to ensure the safety of staff when handling clinical samples that may contain infectious virus. Virus inactivation can be accomplished using various methods (e.g., heat inactivation, chemical inactivation, ultraviolet inactivation, and gamma irradiation [GI]),^2,3^ but special care must be taken to ensure that the inactivation procedure has minimal effect on the analyte being tested.

The rVSVΔG-ZEBOV-GP vaccine candidate is a live recombinant (r) vesicular stomatitis virus (VSV) that has had the VSV G protein removed and replaced with the Zaire Ebola virus (ZEBOV) glycoprotein (GP). Successful phase 1 trials demonstrated general tolerability and robust immune responses to a single vaccination with rVSVΔG-ZEBOV-GP.^4–8^ During the 2013–2016 outbreak, phase 2/3 clinical trials were conducted in the West African countries mainly impacted by the largest recorded ZEBOV outbreak.^9–11^ Efficacy and tolerability were demonstrated in an open-label, cluster-randomized, phase 3 ring vaccination trial conducted during the epidemic in Guinea.^9^ In addition, tolerability and lot consistency, as determined by immunogenicity assessments, were evaluated in a phase 3 study outside of Africa.^12^

During the phase 2/3 rVSVΔG-ZEBOV-GP clinical trials, sera were collected from trial participants in Liberia, Sierra Leone, and Guinea during an active outbreak of Ebola virus disease. Samples were transferred to the United States for testing in the validated Filovirus Animal Nonclinical Group (FANG) human ZEBOV-GP ELISA. At this time, it is not feasible to perform testing using validated assays in the countries where specimens were obtained, and performing immunogenicity testing under maximum containment laboratory (BSL-4) conditions is not practical. To ensure that sera from these trials could be safely handled under BSL-2 conditions, a series of sample-handling procedures was implemented by Merck Sharp & Dohme Corp., a subsidiary of Merck & Co., Inc. (Kenilworth, NJ). These procedures included assuring that no sera from subjects with confirmed or suspected Ebola virus disease were shipped for immunogenicity testing by holding sera for at least 28 days after collection and cross-referencing against reported cases of disease during that period. Gamma irradiation of clinical samples was used to provide an extra level of safety at the testing facility and further reduce risk to laboratory workers. Gamma irradiation has been shown to be a successful tool for inactivating infectious virus for arenaviruses, filoviruses, and influenza viruses.^13–18^ Following GI, serum samples were handled using standard blood-borne pathogen safety procedures under BSL-2.

Compared with other methods of viral inactivation (e.g., detergent, fixation, and heat denaturation), GI has been shown to have minimal impact on antibody conformational integrity, functional activity,^16^ and cytokine immunoreactivity^15^ when conducted under appropriate conditions (i.e., −80°C). However, the impact of the GI treatment on the integrity of antibodies induced by the Ebola GP vaccine has, to our knowledge, not been formally assessed.

Two independent GI studies were prospectively designed to assess the effect of GI at the target dose of 50 kGy on antibody-binding recombinant ZEBOV GP (rGP; by validated ELISA) using human serum samples pre- and postvaccination.

## MATERIALS AND METHODS

### Initial study design and sample preparation.

The initial study was prospectively designed to assess 1) the effect of GI on ZEBOV-rGP–specific antibodies from human serum samples, 2) whether the effect of GI is dependent on the concentration of antibody, 3) whether the measured response is dependent on sample volume and matrix, and 4) whether the effect of GI is dependent on sample location or position in the box (i.e., quadrant within the box).

The study evaluated a panel of 60 individual human serum samples collected from an rVSVΔG-ZEBOV-GP North American phase 1 trial (ClinicalTrials.gov Identifier: NCT02314923),^7^ which spanned the dynamic range of the ELISA (14 negative; 15 low titers [≥ lower limit of quantification {LLOQ} to < 800 ELISA units {EU}/mL], 16 medium titers [≥ 800 to < 1,800 EU/mL], and 15 high titers [≥ 1,800 to ≤ 6,200 EU/mL]). The negative sera were obtained before rVSVΔG-ZEBOV-GP vaccination, and positive sera were obtained 56 days following rVSVΔG-ZEBOV-GP vaccination. In addition to the test samples, the ELISA reference standard, low-quality control (LQC) and high-quality control (HQC) samples (Battelle Memorial Institute, Columbus, OH; Medical Countermeasure Systems Joint Vaccine Acquisition Program, Frederick, MD), and a known monoclonal antibody (mAb) against ZEBOV-GP (mAb KZ52) (IBT Bioservices, Gaithersburg, MD) were included in the panel to further evaluate GI treatment. The reference standard was also used to assess possible dependencies between the effect of GI on the sample volume and the sample location within the cooler and box.

Test samples were thawed at 4°C and aliquoted (500 μL) in quadruplicate in standard, 2.0-mL, sterile, screw-cap, polypropylene tubes. Two tubes from each sample (one to be irradiated and one not) were designated for ELISA testing (Supplemental Table 1). Tubes were separated into 12 numbered boxes (9 rows × 9 columns in standard fiberboard cryovial boxes), with an equal distribution of high-, medium-, and low-titered sera; placebo sera; reference standard; HQC/LQC; and mAb KZ52 across quadrants in boxes (Supplemental Figure 1). The mAb KZ52 and quality control samples were diluted to one concentration (5 μg/mL) in either 1 × PBS (phosphate-buffered saline) or antibody-depleted human serum (ADHS) and aliquoted in different volumes (range 50–800 μL).

To assess whether GI is impacted by the location of the sample within the cooler and box, each box was divided into 9 quadrants (each quadrant of size 3 rows × 3 columns) and the reference standard was placed within each quadrant. Empty spaces within each box contained vials filled with 1 × PBS (approximately 1.8 mL) to mimic a full box configuration but were not subjected to downstream testing. Duplicate boxes were prepared (i.e., containing the same sample aliquots in the exact same positions) so that one complete box would be treated with GI and the second left untreated. The boxes were packaged in four coolers (three boxes per cooler). All samples were refrozen at less than −60°C overnight and shipped on dry ice to the GI location (Sterigenics, Corona, CA), following Category B infectious agent shipping guidelines.

### Follow-up study design and sample preparation.

The follow-up study was prospectively designed with the following objectives: 1) to investigate the unexpected results in the initial study of increased ELISA antibody concentrations for GI-treated, Ebola-GP–negative samples; 2) to evaluate the impact of GI on fold change in antibody response to vaccination (using paired pre- [day 0] and postvaccination [day 56] sera from the same subject); and 3) to evaluate the effect of GI on the freeze–thaw stability profile of human antibodies. To evaluate the first objective, 50 Ebola-GP–negative sera from healthy North American donors with no noted Ebola vaccination history were obtained from a biobroker (Bioreclammation, Inc. [BioIVT], Westbury, NY). To assess the second objective, paired pre- and postvaccination sera were obtained from 20 participants from the phase 1 study (NCT02314923) who had day 0 antibody concentrations above the ELISA LLOQ (36.11 EU/mL) and had no previous vaccination history or suspected ZEBOV exposure. Specimens with concentrations above the LLOQ were chosen so as to quantify fold changes pre- to postvaccination as opposed to estimating the titer at or below LLOQ. For the third objective, 15 serum samples (13 day-56 postvaccination clinical samples from the phase 1 study; two commercially obtained negative samples) were each aliquoted in groups of four. To evaluate the impact, if any, of GI on the stability profile of ZEBOV-GP–specific antibodies through multiple freeze–thaw cycles, two panels underwent two or four freeze–thaw cycles.

As described previously, serum samples were thawed at 4°C and aliquoted into 2.0-mL, sterile, screw-cap, polypropylene tubes; then placed into six numbered boxes (Supplemental Table 1); and frozen at less than −60°C for shipment on dry ice to Sterigenics in Category B packaging. Duplicate boxes were prepared so one complete box would be treated with GI and the second left untreated. Following GI, the boxes were shipped on dry ice to the testing laboratory for freeze–thaw treatments and testing. Briefly, for each freeze–thaw cycle, the serum samples were allowed to thaw at room temperature (≤ 2 hours) and then returned to the freezer (less than −60°C) for at least 4 hours (or overnight) before another freeze–thaw cycle was initiated.

### Sample irradiation.

For both studies, GI was performed by Sterigenics under Good Manufacturing Practice guidelines using a validated GI procedure.

All sera were received frozen and kept frozen overnight in dry ice bins (or less than −60°C) to ensure that samples were completely frozen before and during treatment, and the cold chain was maintained for all prepared samples during transport and GI treatment. The odd-numbered boxes in each study were treated with 50 kGy of GI while on dry ice, whereas the even-numbered boxes were placed in similar validated coolers on dry ice for the same amount of time but not irradiated. The 50-kGy dose used is consistent with routine use to inactivate Ebola in samples existing in BSL-4 laboratories.^13–15^ Dosimeters on each cooler were used to confirm the boxes received the 50-kGy dose. Following GI, all boxes were shipped on dry ice for ELISA testing.

### Filovirus Animal Nonclinical Group human ZEBOV-GP ELISA.

Samples were tested using ELISA at Q^2^ Solutions Vaccines (formerly Focus Diagnostics, San Juan Capistrano, CA).

The indirect FANG human ZEBOV-GP ELISA was validated to measure and quantify antibodies against ZEBOV-GP and is described by Heppner et al.^7^ The assay uses purified ZEBOV rGP as the coating antigen and an enzyme-conjugated anti-human IgG secondary antibody as the reporter or signal system. Briefly, microtiter plates were coated with purified ZEBOV rGP. Samples were then incubated with the rGP-coated wells, allowing ZEBOV-GP–specific antibodies to bind. A serially diluted reference standard, obtained from a pool of vaccinated donors, was also included on each plate along with the HQC and LQC sera. Each well was then incubated with goat anti-human IgG horseradish peroxidase conjugate, which enzymatically reacts with the tetramethylbenzidine substrate. After incubation, the enzymatic reaction was stopped using a sulfuric acid solution, and the optical density was measured on an ELISA plate reader.

Concentrations were calculated from the standard curve using a 4-parameter logistic curve fit and are reported as GP-EU/mL.

### Statistical analyses.

All statistical tests were 2-sided and performed on the natural log–transformed antibody measurements.

In the initial study, the potential dependence on location/position and the effect of GI on the reference standard tested within every quadrant of every box was assessed on the pairwise differences of the natural-log–transformed responses, according to the correspondence between the treated and untreated coolers, using an analysis of variance (ANOVA) model containing fixed terms for “cooler,” “box within cooler,” and “quadrant.”

Given the lack of evidence of meaningful location/position effects identified, the effect of GI on clinical sera was assessed pairwise on the natural logarithm–transformed responses, according to the correspondence between the treated and untreated coolers, using a mixed ANOVA model containing fixed terms for “irradiation,” “titer grouping” (high, medium, low, and negative), and their interaction, and the random term “sample within titer grouping” (as appropriate). The model was restricted to those sample pairs having a determinate result under both test conditions. The effect of GI on the reference standard, quality controls, and mAb spike samples was assessed similarly, but using a mixed ANOVA model containing a single, fixed term for “irradiation.”

The potential for dependence between the effect of GI and sample volume was assessed on the natural logarithm of the effect ratio (i.e., effect of GI on the low-volume/high-volume sample) by sample type (reference standard or mAb spiked into ADHS) using an ANOVA model containing a single, fixed-term for “volume.”

In the follow-up study, the effect of GI on antibody concentrations in Ebola-GP–negative clinical sera was assessed using an exact McNemar’s test. The effect of GI was assessed separately by study day using a paired *t*-test. The fold change (day 56/day 0) was assessed separately by GI treatment using a paired *t*-test, and the fold change (day 56/day 0) was compared with that for the nonirradiated sera using an exact Wilcoxon signed-rank test. In assessing the fold change in response, samples generating results below the ELISA LLOQ were assigned the value of the LLOQ for the purpose of the comparison. Freeze–thaw stability data were analyzed similarly, with differences in titer between paired irradiated and nonirradiated samples compared separately within each freeze–thaw cycle using a paired *t*-test.

## RESULTS

### Antibody concentrations in clinical sera, reference standards, controls, and mAb samples (initial study).

**Figure 1.**
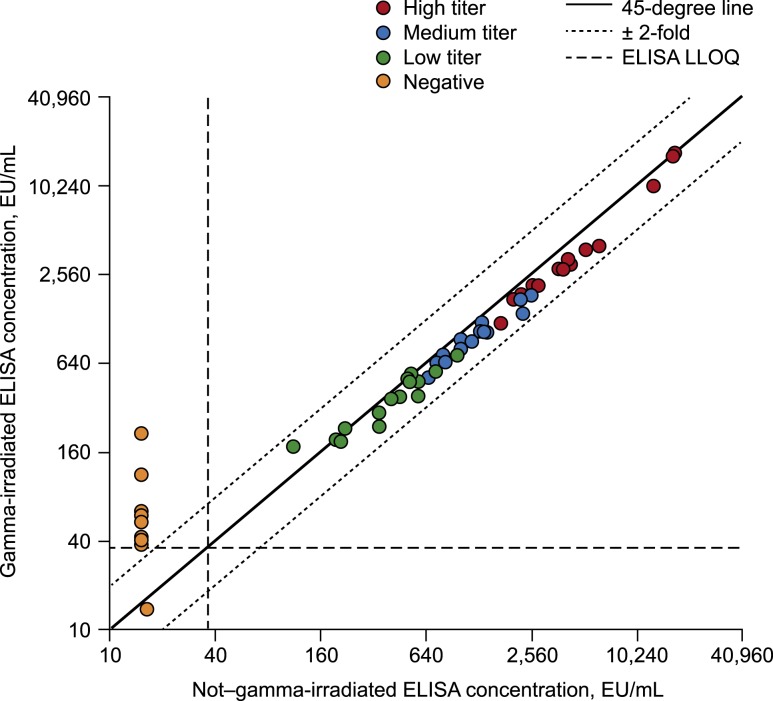
Concordance plot of gamma-irradiated vs. non–gamma-irradiated results for the clinical sera (initial study). EU = ELISA unit; LLOQ = lower limit of quantification.

In the absence of GI, all 14 Ebola-GP–negative (i.e., pre-vaccination) clinical sera tested below the LLOQ (Figure 1, Supplemental Table 2). Following GI, an increase in ELISA concentrations was observed, with 13 of the 14 irradiated sera testing above the LLOQ.

By contrast, in Ebola-GP–positive sera from rVSVΔG-ZEBOV-GP vaccine recipients, a small but statistically significant reduction in detected antibody response was observed following GI (1.21-fold decrease; 95% CI: 1.15- to 1.27-fold) (Table 1, Figure 1). The effect of GI on reducing postvaccination ELISA results was generally consistent across groups based on low, medium, and high titers (Table 1, Figure 1).

**Table 1:** Effect of gamma irradiation on positive clinical sera (ELISA) in the initial study

Titer grouping	*N*	Nonirradiated (EU/mL)		Irradiated (EU/mL)		Concentration ratio (irradiated/nonirradiated)		
		Estimate	95% CI	Estimate	95% CI	Estimate	95% CI	*P*-value
Low	15	387.9	287.2–523.8	350.1	259.3–472.8	0.90	0.83–0.98	0.0117
Medium	16	1,176.5	879.5–1,573.6	932.3	697.0–1,247.0	0.79	0.73–0.86	< 0.0001
High	15	4,395.8	3,255.2–5,936.1	3,476.9	2,574.7–4,695.2	0.79	0.73–0.86	< 0.0001
Combined	46	1,259.3	903.2–1,755.8	1,040.5	746.3–1,450.8	0.83	0.79–0.87	< 0.0001

EU = ELISA unit.

**Figure 2.**
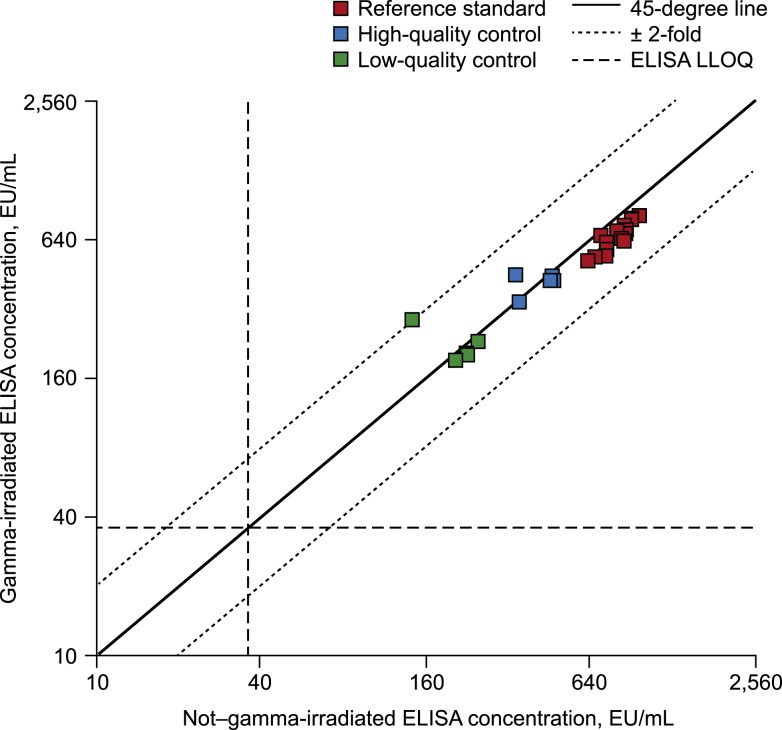
Concordance plot of gamma-irradiated vs. non–gamma-irradiated results for the reference standard and the low-quality control and high-quality control samples. EU = ELISA unit; LLOQ = lower limit of quantification.

**Figure 3.**
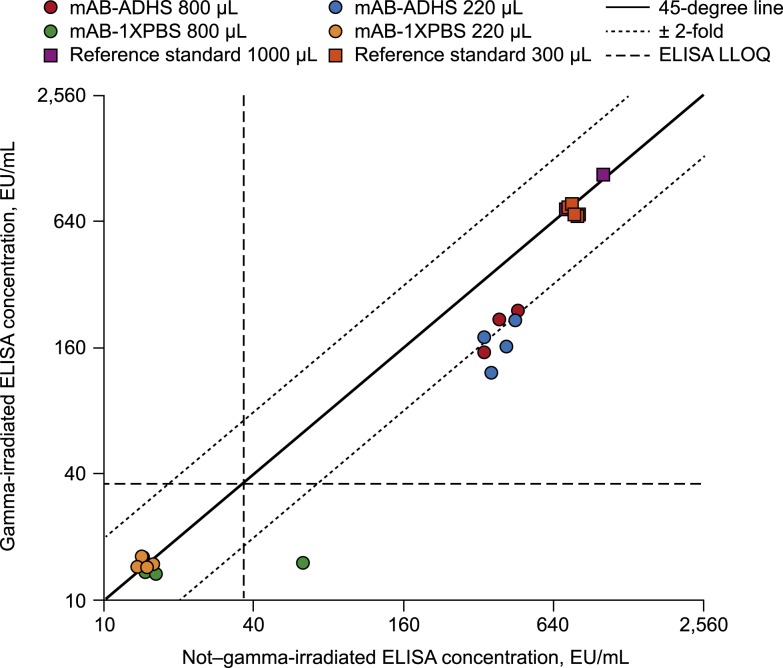
Concordance plot of gamma-irradiated vs. non–gamma-irradiated results for the mAb spike samples and the reference standard evaluated at alternative sample volumes. ADHS = antibody-depleted human serum; EU = ELISA unit; LLOQ = lower limit of quantification; mAb = monoclonal antibody; PBS = phosphate-buffered saline.

Gamma irradiation was also associated with a decrease in measured antibody concentrations for the reference standard and for mAb samples spiked into ADHS (Table 2, Figures 2 and 3). The effect of GI was approximately 1.2-fold for the reference standard (Figure 2) and approximately 2-fold for the KZ52 mAb control spiked into ADHS (Figure 3). The effect of GI on the quality control samples trended toward a lower ELISA result with GI after the exclusion of one HQC sample and one LQC sample that appeared to be outliers (Table 2, Figure 2). In addition, all eight gamma-irradiated mAb samples spiked into PBS (both volumes) tested below the LLOQ, as did seven of the eight samples (both volumes) in the absence of GI (Figure 3). The negative results of the mAb spiked into PBS are most likely due to technical error.

**Table 2:** Effect of gamma irradiation on the reference standard, controls, and mAb spike samples in the initial study (ELISA)

Sample grouping	*N*	Nonirradiated (EU/mL)		Irradiated (EU/mL)		Concentration ratio (irradiated/nonirradiated)		
		Estimate	95% CI	Estimate	95% CI	Estimate	95% CI	*P*-value
Reference standard	23	804.7	763.1–848.7	663.5	629.1–699.7	0.82	0.8–0.85	< 0.0001
1,000 μL	3	844.4	501.4–1,421.8	819.7	486.8–1,380.2	0.97	0.7–1.34	0.7331
300 μL	3	788.9	689.8–902.3	721.7	631.0–825.4	0.91	0.76–1.11	0.1809
HQC	5	417.0	350.1–496.6	419.2	351.9–499.2	1.01	0.83–1.22	0.9428
*HQC	4	437.9	355.2–539.7	411.3	333.7–507.0	0.94	0.89–0.99	0.038
LQC	5	206.8	163.7–261.4	222.4	176.0–281.0	1.08	0.77–1.50	0.5752
*LQC	4	226.5	201.4–254.8	208.3	185.2–234.3	0.92	0.89–0.95	0.0058
mAb-ADHS								
800 μL	4	378.9	286.4–501.4	193.1	145.9–255.6	0.51	0.44–0.59	0.0006
220 μL	4	388.5	285.6–528.5	164.5	120.9–223.7	0.42	0.31–0.58	0.0034

ADHS = antibody-depleted human serum; EU = ELISA unit; HQC = high-quality control; LQC = low-quality control; mAb = monoclonal antibody.

* Excluding one sample that showed an increase in ELISA concentration with gamma irradiation.

### Antibody concentrations and fold changes in clinical sera (follow-up study).

**Figure 4.**
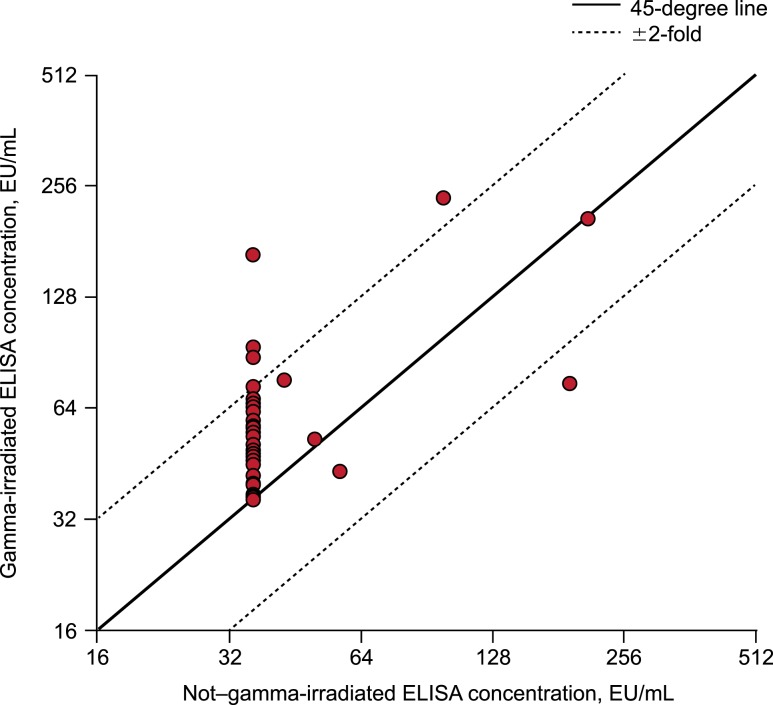
Concordance plot of gamma-irradiated vs. non–gamma-irradiated ELISA antibody concentrations for Ebola-glycoprotein–negative clinical sera (*n* = 50; follow-up study). EU = ELISA unit.

Among the 50 Ebola-GP–negative clinical sera included in the follow-up study, GI was associated with an increase in measured antibody concentration (Figure 4). Consistent with the findings in the initial study, 43 sera tested below the LLOQ in the absence of GI, and 33 of those 43 had a measureable low-level antibody concentration (i.e., ≥ LLOQ) following GI. All seven of the samples that tested above the LLOQ in the absence of GI also tested above the LLOQ with GI.

**Figure 5.**
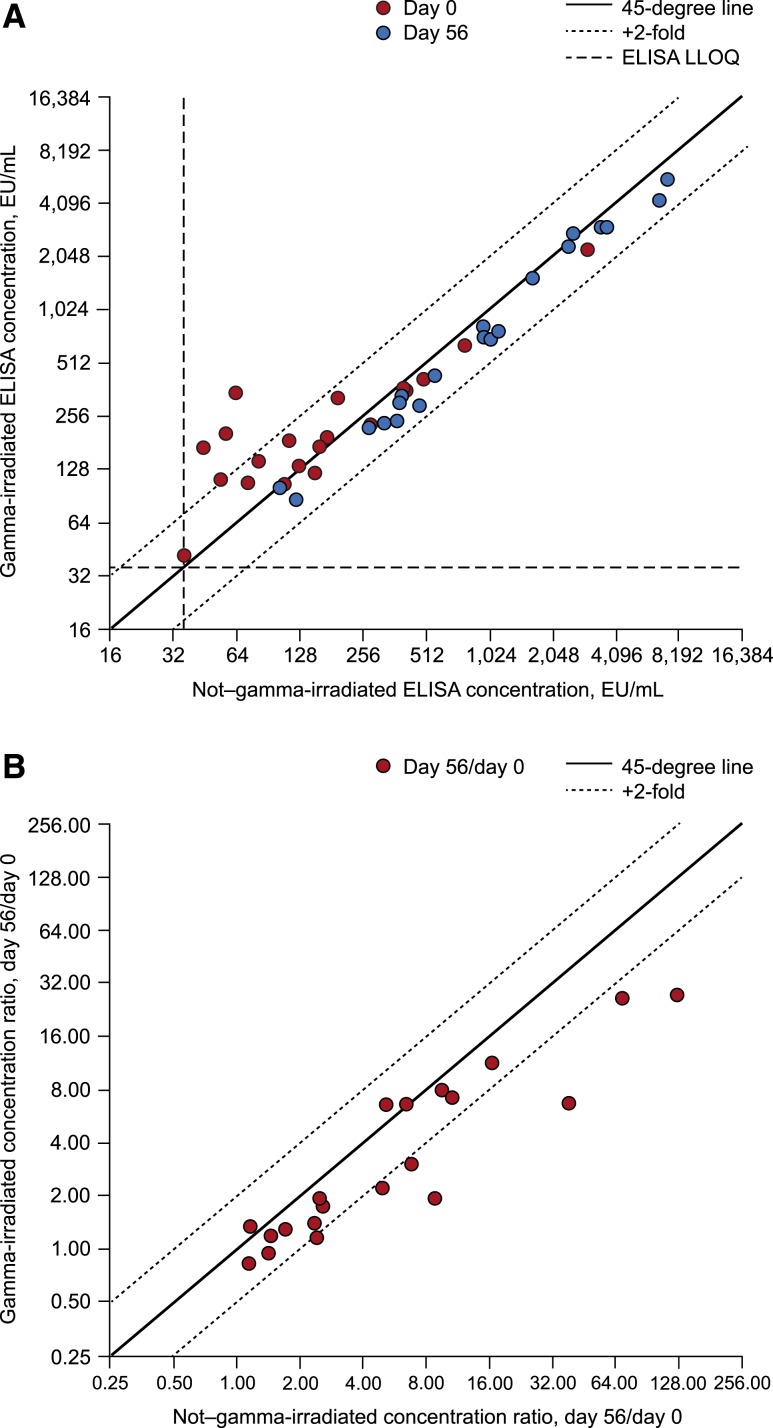
Effect of gamma irradiation on detection of binding (ELISA) antibodies and associated fold rises from baseline in 20 paired day 0 (pre-vaccination) and day 56 (postvaccination) clinical sera. (**A**) ELISA concentration. (**B**) ELISA fold rise. EU = ELISA unit; LLOQ = lower limit of quantification.

ELISA results for the paired pre- and postvaccination sera from 20 study participants who had day 0 antibody concentrations above the LLOQ are summarized by treatment group in Table 3. GI of the pre-vaccination samples was associated with a 36% mean increase (95% CI: 4–78%) in antibody concentrations over concentrations in the absence of GI (Table 3, Figure 5A). Among the 20 matching postvaccination sera, GI was associated with a 21% mean decrease (95% CI: 15–26%) in measured concentrations compared with post-vaccination sera in the absence of GI (Table 3, Figure 5A). As a result, the fold rise in antibody levels after vaccination (i.e., day 56/day 0) was reduced in the presence versus absence of GI (mean 42% reduction; 95% CI: 25–55%) (Table 3, Figure 5B). The geometric mean fold change (95% CI) was 5.58 (2.97–10.59) in the absence of GI and 3.24 (1.95–3.58) following GI (2-sided Wilcoxon signed-rank exact *P* < 0.0001) (Table 3).

**Table 3:** Effect of gamma irradiation on fold change in ELISA antibody concentrations in response to vaccination as measured in the follow-up study

Interval	Nonirradiated (EU/mL)		Irradiated (EU/mL)		Fold difference (irradiated/nonirradiated)	
	GM	95% CI	GM	95% CI	GM	95% CI
Day 0	160.2	96.0–267.5	218.6	148.3–322.1	1.36	1.04–1.78
Day 56	894.0	503.4–1,587.7	707.3	393.3–1,271.8	0.79	0.74–0.85
Fold difference (day 56/day 0)	5.58	2.97–10.49	3.24	1.95–5.38	0.58	0.45–0.75

EU = ELISA unit; GM = geometric mean. Summary statistics on paired pre-vaccination (day 0) and postvaccination (day 56) sera from 20 study participants who had day 0 antibody concentrations above the ELISA lower limit of quantification.

### Sample location/position and volume (initial study), and freeze-thaw cycles (follow-up study).

No statistically significant difference in the effect of GI was detected with regard to the sample volume (neither reference standard 300 and 1,000 μL nor mAb spiked into ADHS 220 and 800 μL; Table 2) or the sample location (i.e., cooler, box within the cooler, or position of the sample within the box), based on data from the initial study (data not shown).

With respect to the effect of freeze–thaw cycles, neither the non-GI nor GI samples showed an appreciable difference in antibody concentration between freeze–thaw cycles 2 and 4 based on data from the follow-up study. The geometric mean fold change in the antibody concentration (95% CI) between cycles 2 and 4 (cycle 4/2) was 1.07 (0.99–1.15) in the absence of GI and 1.04 (0.98–1.11) with GI. The effect of freeze–thaw cycles was comparable in the presence and absence of GI, as the geometric mean ratio of fold changes (GI fold change/non-GI fold change) was 0.96 (0.87–1.05).

## DISCUSSION

We report results from a GI study and confirmatory follow-up study that used a validated clinical ELISA and included clinical trial serum samples from North American subjects vaccinated with the rVSVΔG-ZEBOV-GP vaccine.

Among purported ZEBOV-GP–negative clinical sera (i.e., pre-vaccination), GI was unexpectedly associated with a small increase (approximately 20%) in measured antibody concentrations. The follow-up study confirmed this finding in an additional set of negative clinical sera.

Conversely, among ZEBOV-GP–positive sera (i.e., post-vaccination), GI was associated with a small (approximately 20%) but statistically significant reduction in measured antibody concentrations in both the initial and follow-up studies. A similar reduction in detected antibody concentration for the reference standard was observed, and an even more pronounced effect of GI (2-fold decrease) was observed for mAb spiked into ADHS compared with non-treated samples. The lower antibody concentrations detected in irradiated samples was not unexpected, based on previous publications.^15,16^

There were no apparent differences in the effect of GI with regard to varying sample volume, location (i.e., position of the sample within the box, box within the cooler, or cooler) or the number of freeze–thaw cycles (2 versus 4 cycles). Gamma irradiation had a generally consistent effect across sera grouped by antibody titers.

A potential explanation for the apparent increase in ELISA concentrations in negative samples post-GI is that irradiation, at the high dose of 50 kGy, may lead to breakdown of cellular debris or cause nonspecific antibodies, which are present in all normal human sera, to disintegrate into fragments. In the absence of competition from specific rVSVΔG-ZEBOV-GP antibodies, the debris or fragments could nonspecifically bind to the ZEBOV-GP coating antigen, producing a false-positive signal above the LLOQ. In postvaccination sera, this nonspecific binding would be overwhelmed by specific antibodies that have higher affinity, some of which are also broken down by GI, as evidenced by the finding that specific binding is slightly impaired.

Based on analysis of paired day 0 (pre-vaccination) and day 56 (postvaccination) sera, the fold rise in measured antibody concentration was decreased in a compounded manner by GI because of a combination of the small increase in nonspecific binding in pre-vaccination sera and the decrease in specific binding in postvaccination sera. As a result, a 4-fold rise without GI would appear as a 2.3-fold rise with GI, whereas achieving a > 4-fold rise with GI would require a > 6.9-fold rise without irradiation. Thus, irradiation could reduce the percentage of subjects achieving a > 4-fold rise if a substantial proportion of subjects have a fold rise in the range of 4- to 7-fold without irradiation. The phenomenon of increased pre-vaccination and decreased postvaccination ELISA concentrations should be considered when presenting results from irradiated specimens, especially with respect to fold rise and seroresponse.

Limitations of this study include the use of specimens from North American subjects, who potentially have minimal baseline antibody levels. The target demographic for the vaccine will likely be African subjects living in regions where Ebola circulates, and the authors acknowledge that a fold increase in ELISA titers could be affected more in this target demographic than was observed with North American subjects because of preexisting antibodies or higher incidence of nonspecific binding in ELISA (unpublished results). This study could not use human serum samples collected from patients who may have been infected with Ebola or were confirmed positive for wild-type Ebola virus because this study was conducted in BSL-2 laboratories. Future studies that include subjects who may have been infected with Ebola or were confirmed positive for wild-type Ebola virus can be performed if appropriate BSL laboratories are available.

Despite the observation that irradiation impacts antibody detection in pre- and postvaccination clinical sera, as measured by ELISA, a correction factor is not recommended for determining the final antibody concentrations of gamma-irradiated clinical sera because the presence and magnitude of the effect for pre-vaccination sera appears to be sample specific. Moreover, because the effect of GI was consistent through the range of antibody concentrations and all samples within a given study will be treated in the same manner, the effect of GI should not bias treatment comparisons within a study.

Overall, GI appears to be a viable method for treating sera collected in regions where filoviruses are of concern, with measurable impact on immunogenicity analyses that should be accounted for in interpretation of the results. The decreased probability of transmitting viable Ebola virus to testing personnel far outweighs the small effect on the antibody titer. Gamma irradiation is a useful tool to irradiate clinical sera in areas where rare or unknown pathogens are a concern; however, the effect of GI on pre- and postvaccination results must be taken into consideration.

## Supplementary Files

Supplemental tables and figure
