# Supplementary material for: Effect of Gamma Irradiation on the Antibody Response Measured in Human Serum from Subjects Vaccinated with Recombinant Vesicular Stomatitis Virus–Zaire Ebola Virus Envelope Glycoprotein Vaccine
Source: Am J Trop Med Hyg. 2019 Jun 3;101(1):207–13. doi: 10.4269/ajtmh.19-0076 (PMC6609194; doi:10.4269/ajtmh.19-0076)

SUPPLEMENTARY MATERIAL

SUPPLEMENTARY TABLE 1. Study design: test samples for the initial study and follow-up study

Box	Cooler	Gamma irradiation dose	Shared aliquots of test samples in treated and non-treated boxes
**Initial study**			
1	1	50 kGy	20 clinical sera
2	2	NA	11 reference standard samples
			8 mAb samples
			6 quality-control samples (3 low/3 high)
5	1	50 kGy	20 clinical sera
6	2	NA	13 reference standard samples
			8 mAb samples
			4 quality-control samples (2 low/2 high)
9	3	50 kGy	20 clinical sera
10	4	NA	5 reference standard samples

**Follow-up study**			
1	1	50 kGy	50 negative human sera in each box (biobroker)
2	2	NA	
5	1	50 kGy	A. 20 V920-004 sample pairs (day 0/day 56) for a total of 40 vials in box 5 and 40 vials in box 6.<br>B. 15 V920-004 and biobroker sera for stability aliquoted in 8 identical panels/box (ELISA Panels 1–4). 30 vials in box 5 and 30 vials in box 6<br>Overall total of 70 vials in each box
6	2	NA	

mAb = monoclonal antibody; NA = not applicable.

SUPPLEMENTARY TABLE 2. Effect of gamma irradiation on negative clinical sera (ELISA) in the initial study

	Sample														
Treatment	N1	N2*	N3	N4	N5	N6	N7	N8	N9	N10	N11	N12	N13	N14	N15
Not irradiated	<36.11	NA	<36.11	<36.11	<36.11	<36.11	<36.11	<36.11	<36.11	<36.11	<36.11	<36.11	<36.11	<36.11	<36.11
Irradiated	55.98	NA	64.26	54.04	40.30	60.80	217.20	<36.11	54.96	62.38	38.87	42.71	61.59	37.57	113.40

* Sample N2 was from a rVSVΔG-ZEBOV-GP recipient.

SUPPLEMENTARY FIGURE 1. Layout of samples in each box for the initial study. Empty spaces were filled with 1xPBS, which was not tested. Coolers 1 and 3 were treated with gamma irradiation and contained all odd-numbered boxes. Coolers 2 and 4 were not treated with gamma irradiation and contained all even-numbered boxes. Boxes 1, 2, 5, 6, 9, and 10 contained sample aliquots for ELISA.

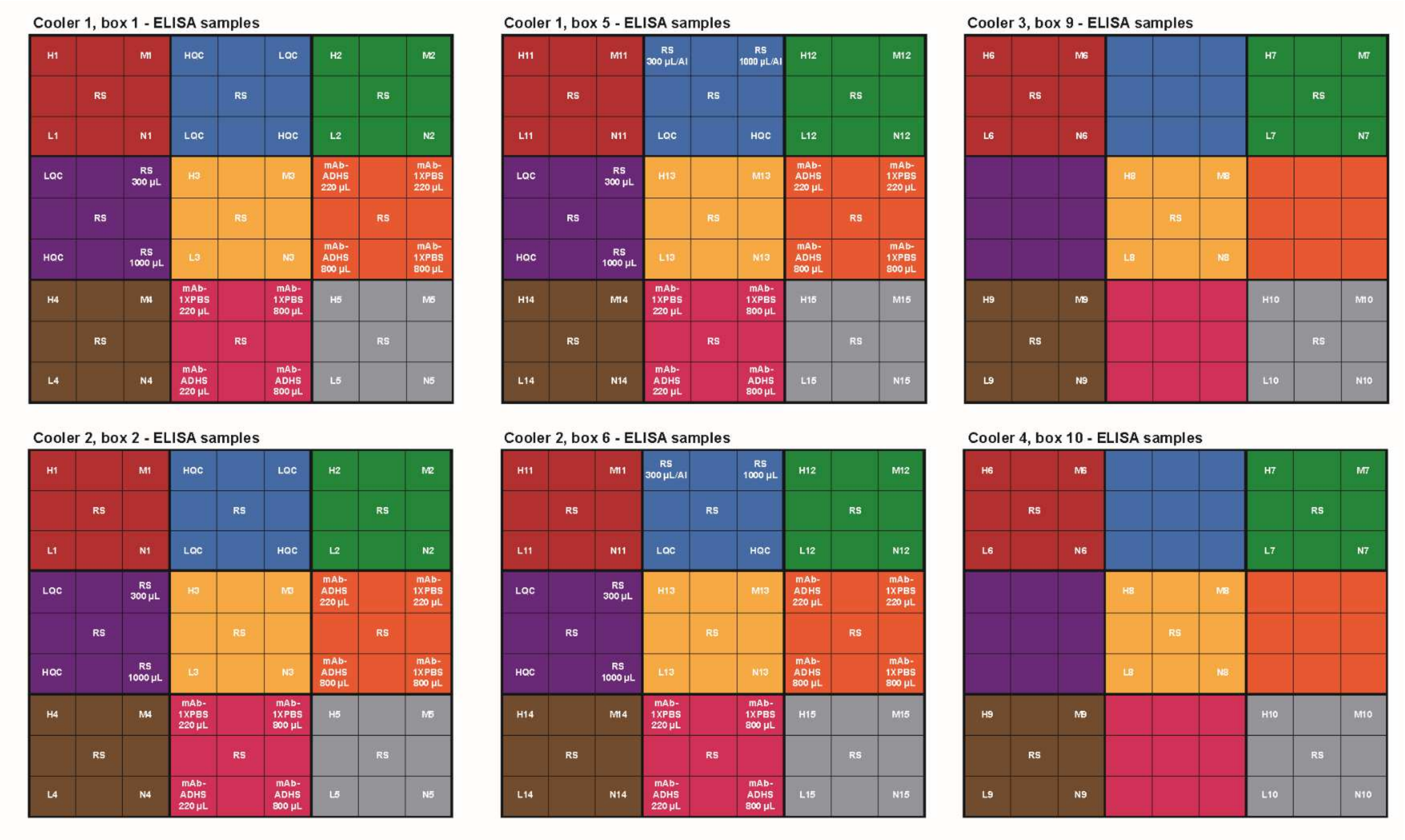

Supplement: Supplementary file 1 [file tpmd190076.SD1.pdf]
